# Numerical Simulation and Optimization of Highly Stable and Efficient Lead-Free Perovskite FA_1−x_Cs_x_SnI_3_-Based Solar Cells Using SCAPS

**DOI:** 10.3390/ma15144761

**Published:** 2022-07-07

**Authors:** Hussein Sabbah, Jack Arayro, Rabih Mezher

**Affiliations:** College of Engineering and Technology, American University of the Middle East, Kuwait; jack.arayro@aum.edu.kw (J.A.); rabih.mezher@aum.edu.kw (R.M.)

**Keywords:** solar cell, photovoltaics, thin films, SCAPS simulation, lead-free perovskite, tin-based perovskite, power conversion efficiency, electron transport layer

## Abstract

Formamidinium tin iodide (FASnI_3_)-based perovskite solar cells (PSCs) have achieved significant progress in the past several years. However, these devices still suffer from low power conversion efficiency (PCE=6%) and poor stability. Recently, Cesium (Cs)-doped Formamidinium tin iodide (${\mathrm{FA}}_{1-x}{\mathrm{Cs}}_{x}{\mathrm{SnI}}_{3})$ showed enhanced air, thermal, and illumination stability of PSCs. Hence, in this work, ${\mathrm{FA}}_{1-x}{\mathrm{Cs}}_{x}{\mathrm{SnI}}_{3}$ PSCs have been rigorously studied and compared to pure FASnI_3_ PSCs using a solar cell capacitance simulator (SCAPS) for the first time. The aim was to replace the conventional electron transport layer (ETL) TiO_2_ that reduces PSC stability under solar irradiation. Therefore, ${\mathrm{FA}}_{1-x}{\mathrm{Cs}}_{x}{\mathrm{SnI}}_{3}$ PSCs with different Cs contents were analyzed with TiO_2_ and stable ZnOS as the ETLs. Perovskite light absorber parameters including Cs content, defect density, doping concentration and thickness, and the defect density at the interface were tuned to optimize the photovoltaic performance of the PSCs. The simulation results showed that the device efficiency was strongly governed by the ETL material, Cs content in the perovskite and its defect density. All the simulated devices with ZnOS ETL exhibited PCEs exceeding 20% when the defect density of the absorber layer was below $10^{15}{\;\mathrm{cm}}^{-3}$, and deteriorated drastically at higher values. The optimized structure with ${\mathrm{FA}}_{75}{\mathrm{Cs}}_{25}{\mathrm{SnI}}_{3}$ as light absorber and ZnOS as ETL showed the highest PCE of 22% with an open circuit voltage $V_{\mathrm{oc}}$ of 0.89 V, short-circuit current density $J_{\mathrm{sc}}$ of $31.4\;\mathrm{mA}\cdot {\mathrm{cm}}^{-2}$, and fill factor FF of 78.7%. Our results obtained from the first numerical simulation on Cs-doped FASnI_3_ could greatly increase its potential for practical production.

## 1. Introduction

Due to their carbon footprints, traditional sources of energy are major contributors to climate change and global warming, representing direct threats to the current century. Over the last two decades, there has been an international movement towards substituting the use of fossil fuels with other sources that are renewable, environmentally safe and sustainable. One of the most prominent sources of renewable energy is solar energy. Solar energy can be harnessed by photovoltaic panels, which are an alternative method to generate electricity [1,2,3]. The first generation of solar cells was silicon-based with a high-power conversion efficiency (PCE), reaching 25%. However, the high manufacturing cost of this kind of panel made them inaccessible to the public and they were only used in specific industrial fields, such as in the space industry. To overcome the cost issue, another generation of solar cells were developed: lead (Pb)-based perovskite solar cells (PSCs). Over the past decade, extensive work has led to rapid improvement in the efficiency of this type of cell, from 3.8% to over 25.5% [4,5].

In addition to their low manufacturing cost, and their high efficiency, lead-based perovskite solar cells are found to have low exciton-binding energies, high optical absorption coefficients, long diffusion lengths and tunable bandgaps [6,7,8,9]. In parallel, one notices from the literature the rise of a specific lead-based PSC, more precisely, lead halide perovskites. Due to their high absorption coefficient and large diffusion length, the latter PSCs are found to have additional applications, such as in photocatalysis [10], in lasers [11,12] and in LEDs [13,14].

Despite the previously mentioned advantages, lead is known to be a toxic material and degradable. A solution for this latter drawback is to substitute lead with other materials (Tin (Sn)), leading to the appearance of a new era of photovoltaic cells, in particular, Sn-based perovskite solar cells. Indeed, since Sn and Pb are both in group 14 of the periodic table and have similar ion radii [15,16], tin is considered an adequate alternative to Pb. Sn-based perovskites are used since they allow tuning of the band gap by simple composition substitution [17,18]. This is an additional reason why they are considered in this study [19].

In solar cell applications, three major typical Sn-based perovskites are used: Methylammonium tin iodine perovskites (MASnI_3_), Formamidinium tin iodine perovskites (FASnI_3_), and Cesium tin iodine perovskites (CsSnI_3_) [20]. Despite being more stable than MASnI_3_ and FASnI_3_, CsSnI_3_ posesses the lowest PCE among Sn-based perovskite solar cells [21,22,23]. To circumvent the PCE deficiency, Kim et al. [24] investigated the effect of incorporating additives (such as SnF_2_, SnCl_2_ and SnBr_2_) to the CsSnI_3_ structure. It has been found that SnBr_2_ is the most convenient additive, increasing the PCE by 4.3%, while providing even better stability for Sn-based perovskites.

Methylammonium tin iodine MASnI_3_ is known to have a stable structure [25], an improved photo responsiveness [26], a long carrier-diffusion length [27] and a superior carrier mobility [28]. However, the power conversion efficiency of MASnI_3_ solar cells is relatively low compared to Pb-based perovskite solar cells [29]. Although they are both stable in an inert atmosphere, FASnI_3_ is found to be more stable than MASnI_3_ [30]. Therefore, high-performant Sn-based perovskite solar cells mainly adopt FASnI_3_, instead of MASnI_3_, despite the fact that both materials are sensitive to air [31]. An extensive work aiming to stabilize FASnI_3_ has been done [32], all while maintaining outstanding photovoltaic properties. This work is based on introducing antioxidant additives, such as hydroxybenzene sulfonic acid [33], guanidinium [34], GeI_2_, and SnF_2_ [35], to the FASnI_3_.

Previously cited works herein emphasized the common issues and limitations that arise in employment of pure FASnI_3_ perovskites [36], namely, oxidation and crystal structure deviation, and represent doping as a solution to the stated problems. In particular, extensive work done by [19,36,37,38,39,40,41,42] focused on Cesium (Cs) as a promising element to be used for FASnI_3_ doping. In fact, Cs added to FASnI_3_ can act as a reduction agent to limit the oxidation of ${\mathrm{Sn}}^{2+}$ to ${\mathrm{Sn}}^{4+}$. Moreover, substituting FA with Cs, of smaller atomic radius than FA, leads to lattice contraction, reducing the free energy of the structure, increasing stability and preventing deviation of the crystal structure. Furthermore, ${\mathrm{FA}}_{1-x}{\mathrm{Cs}}_{x}{\mathrm{SnI}}_{3}$ is found to have better air, illumination and thermal stability, as well as better photovoltaic properties, such as trap state density and light response range [19,36]. Also, Cs-doped FASnI_3_ increases electron mobility by a factor of three [36]. In addition to the above-mentioned advantages, the PCE of the ${\mathrm{FA}}_{1-x}{\mathrm{Cs}}_{x}{\mathrm{SnI}}_{3}$ shows a 63% increase compared to the pure device (from 3.74% to 6.08%), due to the improved quality of the ${\mathrm{FA}}_{1-x}{\mathrm{Cs}}_{x}{\mathrm{SnI}}_{3}$ film [36]. This experimental result is still far from the PCE of 25% recorded by lead-based perovskite solar cells [43], hence, additional studies are still needed for further improvement in the PCE.

This work aimed to suggest possible optimization routes for efficiency improvements of the stable ${\mathrm{FA}}_{1-x}{\mathrm{Cs}}_{x}{\mathrm{SnI}}_{3}$ perovskite solar cell, by analyzing various device parameters using the solar cell capacitance simulator (SCAPS-1D) [44]. Recent studies confirm the rise of SCAPS-1D as a powerful tool in the advancement of solar cell technology.

The studies show good agreement between the simulation results and the experimental data, indicating the reliability of SCPAS software [45,46]. SCAPS simulator has exceptional features, including, but not limited to, simulating up to seven layers, calculating many parameters, like spectral response, energy bands, J-V curve, and defect density, by solving just three basic semiconductor equations. It is user friendly and may be executed in both dark and light atmospheres [47,48,49].

In this paper, a comparative study between pure FASnI_3_ and ${\mathrm{FA}}_{1-x}{\mathrm{Cs}}_{x}{\mathrm{SnI}}_{3}$ is presented. The photovoltaic performances of these two absorbers were investigated by coupling them with two different materials as ETL: TiO_2_ and ZnOS, which recently showed promising results [30,50,51,52,53,54].

The impact of the defect density, thickness, acceptor doping concentration, electron and hole mobility of the absorber layer and the interface defect density between the perovskite from one side and the HTL (Hole Transport Layer)/ETL (Electron Transport Layer) from the other side, on the overall performance of the proposed device was studied. It was proven that an optimum ${\mathrm{FA}}_{1-x}{\mathrm{Cs}}_{x}{\mathrm{SnI}}_{3}$ device could have a simulated power conversion efficiency PCE of 22%.

## 2. Materials and Methods

In the present study, a numerical simulation was conducted on ${\mathrm{FA}}_{1-x}{\mathrm{Cs}}_{x}{\mathrm{SnI}}_{3}$, considered the light absorber, using SCAPS 3.8, which is a 1D solar cell simulation software developed at the Department of Electronics and Information Systems (ELIS) of the University of Gent, Ghent, Belgium. [44]. SCAPS allows simulation of multilayer solar cells (up to seven layers). In SCAPS, one can calculate and observe many electrical characteristics and parameters, such as power conversion efficiency PCE, hetero-junction energy band structure, current-density (J-V) curve, open circuit voltage $V_{\mathrm{oc}}$, short circuit $J_{\mathrm{sc}}$, quantum efficiency (QE), current density, fill factor FF, amongst others. SCAPS solves with an adapted algorithm, the Poisson’s equation, Equation (1) and the continuity equation of both charge carriers: electron Equation (2) and hole Equation (3)

(1) $$\frac{d}{dx}\left(-\varepsilon \left(x\right)\frac{d\psi }{dx}\right)=q\left[p\left(x\right)-n\left(x\right)+N_{D}^{+}\left(x\right)-N_{A}^{-}\left(x\right)+p_{t}\left(x\right)-n_{t}\left(x\right)\right]$$

(2) $$\frac{dp_{n}}{dt}=G_{p}-\frac{p_{n}-p_{n0}}{\tau_{p}}+p_{n}\mu_{p}\frac{d\xi }{dx}+\mu_{p}\xi \frac{dp_{n}}{dx}+D_{p}\frac{d^{2}p_{n}}{dx^{2}}$$

(3) $$\frac{dn_{p}}{dt}=G_{n}-\frac{n_{p}-n_{p0}}{\tau_{n}}+n_{p}\mu_{n}\frac{d\xi }{dx}+\mu_{n}\xi \frac{dn_{p}}{dx}+D_{n}\frac{d^{2}n_{p}}{dx^{2}}$$

**Figure 1:**
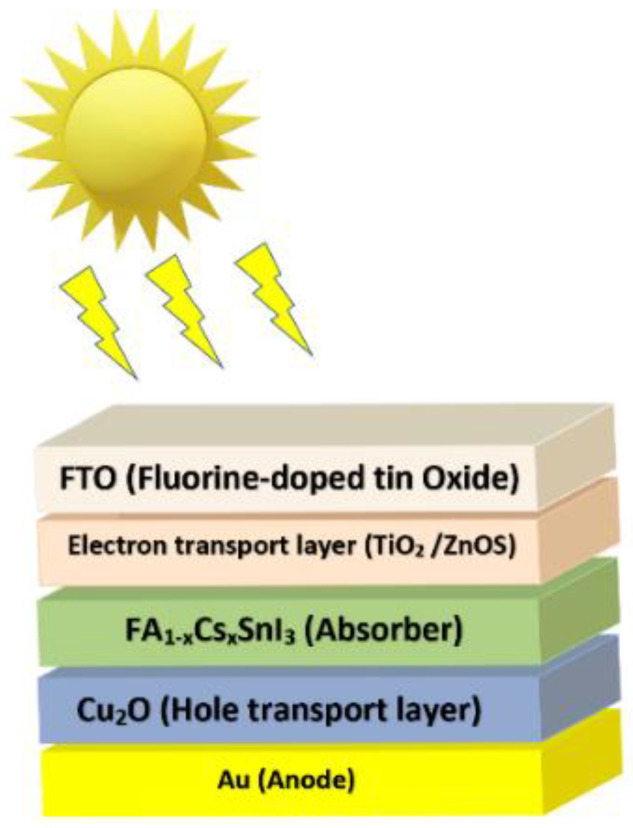
Schematic diagram of lead-free ${\mathrm{FA}}_{1-x}{\mathrm{Cs}}_{x}{\mathrm{SnI}}_{3}$-based PSC structure.

To simulate the device a *n* − *i* − *p* configuration of FTO/ETL/${\mathrm{FA}}_{1-x}{\mathrm{Cs}}_{x}{\mathrm{SnI}}_{3}$/Cu_2_O/Au is considered (Figure 1); where the proportion *x* varies between: 0.00, 0.10, 0.15 and 0.25.

The simulation was performed at a temperature of 300 K under standard illumination of $1000\;W/m^{2}$, and an air mass of AM 1.5 G. As shown in the figure, the absorber layer was placed between the HTL and ETL layers. As a front contact and back metal, Fluorine-doped tin oxide (FTO) and back metal gold (Au) were used, respectively. For every structure, the considered HTL was Cu_2_O, while the ETL material alternated between ZnOS and TiO_2_. A comparison between the two latter materials was performed.

**Figure 2:**
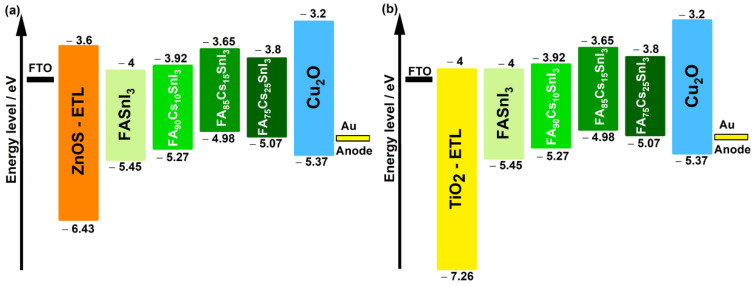
Band alignment between ${\mathrm{FA}}_{1-x}{\mathrm{Cs}}_{x}{\mathrm{SnI}}_{3}$ perovskites and (**a**) ZnOS ETL, (**b**) and TiO_2_ ETL.

Figure 2 illustrates the energy level diagram of the considered materials in the device structure. Figure 2a,b include TiO_2_ and ZnOS as ETL layers, respectively.

The electrical and optical parameters implemented in the simulation, extracted from both experimental and theoretical works [55,56,57,58,59,60,61,62,63,64], are grouped in Table 1, Table 2 and Table 3. Conduction band minima and band gap for pure FASnI_3_ and the ${\mathrm{FA}}_{1-x}{\mathrm{Cs}}_{x}{\mathrm{SnI}}_{3}$ perovskites were extracted from the experimental studies performed by M. D. McGehee et al. [19].

Different values, such as defect density, thickness, shallow acceptor, electron and hole mobility of the absorber layer and the interface defect between the perovskite from one side and the HTL/ETL from the other side of the absorber layer, were varied to obtain an optimized result and to study their impacts on device performance. Due to their correspondingly high PCEs, the thickness chosen for FTO, ETL, and Cu_2_O were, respectively, 0.40 μm, 0.05 μm, and 0.35 μm (Table 1 and Table 2).

## 3. Results and Discussion

In this part, the results are presented. First, a preliminary study on the structure of the solar cell and its effect on performance was conducted. As a conclusion of this study, the optimal structure was chosen and further investigations were based on it. In particular, the effect of the absorber layer regarding defects density $N_{t}$ and acceptor doping concentration $N_{A}$, the optimization of defect interface of the solar cell and absorber layer thickness, as well as the effect of electron and hole mobility of the absorber on solar cell performance, were considered.

### 3.1. Effect of Structure on Solar Cell Performance

In order to assess the effect of the structure on the performance of PSCs two aspects were considered; first, two types of ETL layer were tested, TiO_2_ and ZnOS, then, different systems were considered, while varying Cs content, at 10%, 15% and 25%. These studied aspects would then be compared to the pure FASnI_3_ structure.

Numerous studies emphasize the importance of the energy level alignment between the absorber (PSC) and the ETL layer (TiO_2_ or ZnOS) [65,66,67,68]. This energy level alignment is represented by CBO, the conducting band offset, which is the electron affinity difference between the ETL and the absorber (perovskite) (Equation (4)):

(4) $$CBO=\chi_{Absorber}-\chi_{ETL}$$

Therefore, interface engineering and control at the ETL-perovskite interface is crucial for addressing the CBO and achieving high-efficiency planar PSCs [32,33]. Moreover, one of the challenges in PSCs is the recombination loss across the interfaces, especially at the ETL/absorber, which can lower the voltage [35]. In addition to the band alignment, an optimal ETL material should also have high electron mobility and excellent photochemical stability under UV light. To this end, the above-mentioned properties were compared between the ZnOS and TiO_2_ ETL layers.

**Figure 3:**
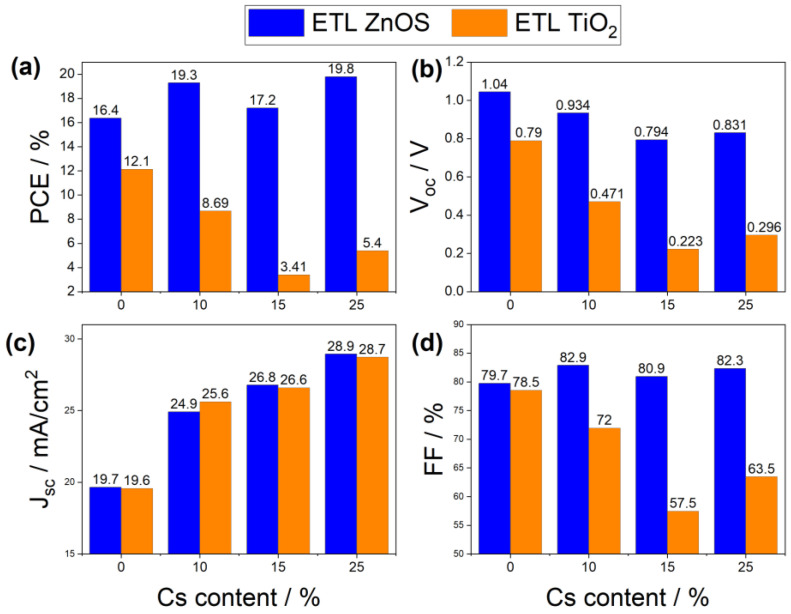
Behavior of (**a**) PCE, (**b**) $V_{\mathrm{oc}}$, (**c**) $J_{\mathrm{sc}}$, and (**d**) FF for absorber as a function of Cs content and different ETL materials; ZnOS is in blue and TiO_2_ is in orange.

For best assessment of the Cs content and the ETL material choice, four different parameters were investigated: the PCE, the voltage open circuit ($V_{\mathrm{oc}}$), the short-circuit current density ($J_{\mathrm{sc}}$) and the fill factor (FF). Figure 3 illustrates the obtained behavior of the above-mentioned parameters for different Cs contents and ETL materials. A general overview of Figure 3 clearly indicates that, regardless of the Cs content and the CBO between the perovskite and the ETL, the devices with ZnOS as ETL surpassed those with TiO_2_ as ETL. In fact, the difference of mobility of electrons in the two ETL materials could be a direct reason for this discrepancy. As shown in Table 1, the ZnOS had an electron mobility five times higher than that of TiO_2_. All simulated solar devices with ZnOS as ETL showed high PCE, exceeding 16%, with large $V_{\mathrm{oc}},$ exceeding 0.79 V, and high FF of 80%. However, the ultimate PSC with TiO_2_ as ETL only showed a PCE of 12%. The inferior electron mobility of TiO_2_ compared to that of the perovskite could lead to a significant charge recombination in the ETL, thus resulting in unbalanced charge transfer and, consequently, low power convergence efficiency [68].

Another crucial factor behind the outperformance of the solar cells with ZnOS as ETL was the enhanced band alignment of the ZnOS against the perovskite. In addition, Figure 3 reveals an interesting difference between the photovoltaic performance of the devices having the same ETL but different perovskites with different Cs contents. The CBO between the perovskite and the ETL was one of the reliable reasons for this behavior.

The values of the CBO (found from Equation (4) and Figure 2) for ETL ZnOS and TiO_2_, for different Cs content are grouped in Table 4.

From Table 4, one can notice that the CBO for all the Cs-doped FASnI3 devices was smaller than that of the pure FASnI3 cell, all while keeping positive values for the PSC with ZnOS as ETL. A positive CBO indicated a spike structure formed at the ETL/absorber layer interface which could act as a barrier for photo-generated electron flow and prevent electrons from reaching the ETL-absorber interface. This barrier endowed enhanced photo-generation of free charge carriers, and would suppress the recombination rate at the interface and reduce the $V_{\mathrm{oc}}$. Consequently, this spike structure favored increase in the efficiency of power conversion of the solar cells with ZnOS as ETL.

When TiO_2_ was used as ETL, it can be noticed from Table 4, that the values of the CBO were always negative in the cases of Cs-doped FASnI_3_, and zero in the case of the pure structure. A negative CBO indicated that the CB level of ETL was lower than that of the perovskite, resulting in the formation of an energy cliff at the ETL-perovskite interface.

**Figure 4:**
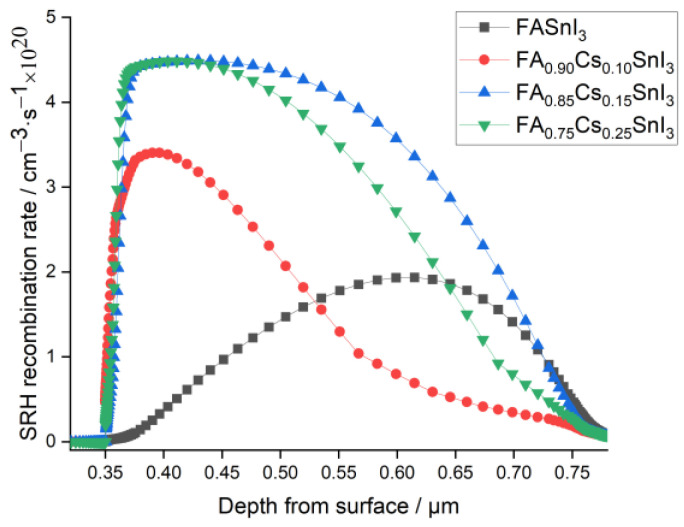
Effect of the Cs content in the absorber with TiO_2_ ETL on the recombination rate with depth from the surface.

Hence, it can be noticed from Figure 4, representing the SRH recombination rate for the studied systems through the layer, that the electron holes recombination rate increased, causing a drop in the $V_{\mathrm{oc}}$ and the PCE levels, as indicated in Figure 3.

Thus, when TiO_2_ was used as ETL, Cs doping did not help in enhancing the performance of the PSC. On the contrary, Cs doping was found to deteriorate the photovoltaic properties, and decreased the PCE from 12.1%, in the case of pure FASnI_3_, to 3.41%, when the Cs doping was 15%.

**Figure 5:**
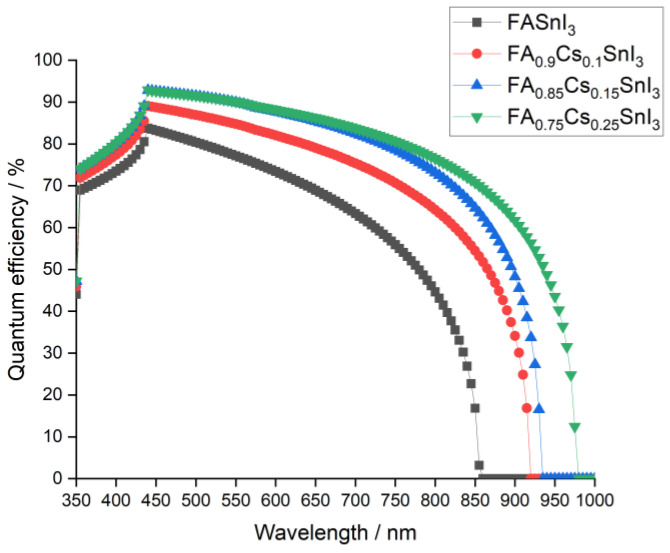
Quantum efficiency of the simulated device with ZnOS ETL and different Cs content.

From Figure 3, it can be noticed that $J_{\mathrm{sc}}$ increased with doping for all devices. This behavior was attributed to the smaller band gaps obtained when the Cs content was more and more enriched, as shown in Table 1. Indeed, as the Cs concentration increased the quantum efficiency, illustrated in Figure 5, reached higher peaks, indicating that more photoelectrons would be generated and, thus, a higher $J_{\mathrm{sc}}.$

In brief, regardless of the Cs content, ZnOS was found to provide better photovoltaic properties compared to TiO_2_. Mainly, this was due to much higher electron mobility and better band alignment with the perovskite. Figure 3 revealed that doping the structure with Cs had great benefits in enhancing the properties of PSC with ZnOS ETL. In fact, the PCE in the case of the 25% Cs-doped structure increased by 3.4% with respect to the case of pure FASNI_3_. In addition, it is worth mentioning here that the PCE was proportional to $V_{\mathrm{oc}}$ and $J_{\mathrm{sc}}$ [69]. For that reason, different behavior of the PCE could be witnessed, which explained the drop of 2.1% in the PCE between the two-doping contents of 10% and 15%.

Comparing the results plotted in Figure 3, with the CBO values of Table 4**,** one can notice that the best structure corresponded to a Cs doping concentration of 25%, with a CBO of 0.2 eV, PCE of 19.8%, $V_{\mathrm{oc}}=0.831\;V$, $J_{\mathrm{sc}}=28.9\;\mathrm{mA}/{\mathrm{cm}}^{2}$ and FF=82.3%.

The latter result is in accordance with previous studies [30] suggesting ZnOS as a promising ETL to replace TiO_2_. Therefore, in the following stages of this study, ZnOS as ETL with a FASNI_3_ absorber doped with Cs at 25% (FA_0.75_Cs_0.25_SnI_3_) was adopted.

### 3.2. Effect of Absorber Layer Defects Density $N_{t}$ and Acceptor Doping Concentration $N_{A}$

In addition to the importance of choosing the adequate ETL material and the structure of the absorber layer, defects density of this layer $N_{t}$ and acceptor doping concentration $N_{A}$ are also of high relevance. On one hand, a high $N_{t}$ means more defects, leading to a high recombination rate of the carrier that affects the device output [70,71]. On the other hand, it has been found that, as the acceptor doping concentration $N_{A}$ increases, the overall performance of solar cells improves [58].

**Figure 6:**
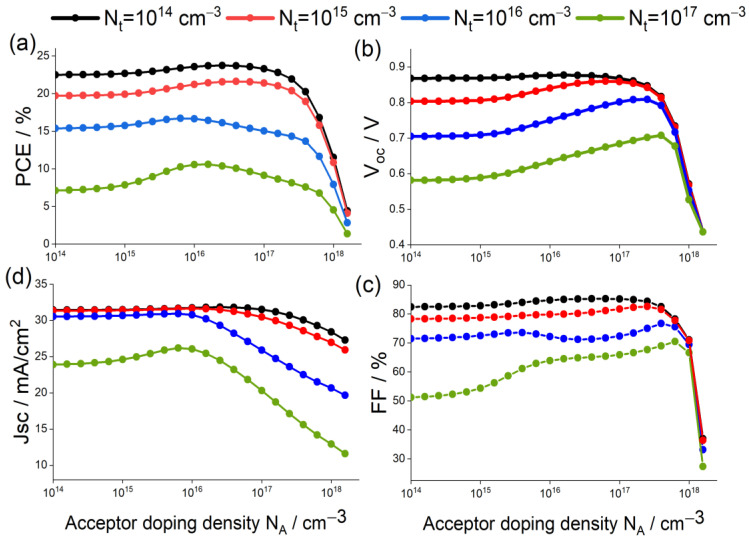
Variation in (**a**) PCE, (**b**) $V_{\mathrm{oc}}$, (**c**) FF, and (**d**) $J_{\mathrm{sc}}$ for absorber with different defect density and acceptor doping concentration.

**Figure 7:**
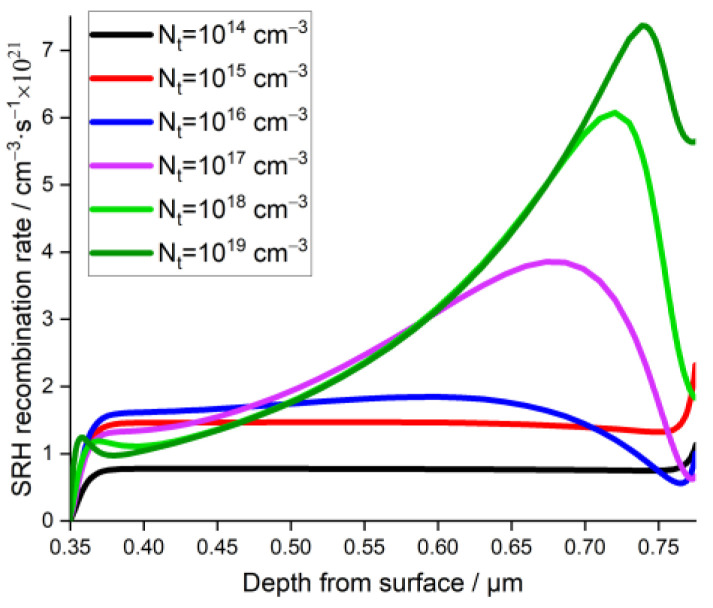
Effect of the absorber layer defect density on the recombination rate with depth from the surface.

Figure 6 represents the evolution of the PSC photovoltaic parameters as function of $N_{A}$, for different values of $N_{t}.$ From the latter figure, one can notice that regardless of the $N_{A}$ values, PCE and $V_{\mathrm{oc}}$ were almost identical for $N_{t}=10^{14}$ and$\;10^{15}{\;\mathrm{cm}}^{-3},$ then they drastically decreased when $N_{t}$ was greater than $10^{15}{\;\mathrm{cm}}^{-3}.$ This result could be related to the fact that the SRH recombination rate exhibited higher values after a threshold value of $N_{t}=10^{15}{\;\mathrm{cm}}^{-3}$, as shown in Figure 7.

In addition, it can be noticed from Figure 6 that for all the considered values of $N_{t}$, $V_{\mathrm{oc}}$ and $J_{\mathrm{sc}}$ increased until they reached their maxima at $N_{A}=6.3\times 10^{16}{\;\mathrm{cm}}^{-3}$ and $N_{t}=1\times 10^{14}{\;\mathrm{cm}}^{-3},$ then abruptly decreased. The fill factor FF and the power convergence efficiency PCE of the solar cell devices followed this trend. In fact, when the acceptor doping concentration increased, the Fermi energy level of the hole decreased and, hence, $V_{\mathrm{oc}}$ increased. Another aspect is that, as the acceptor doping concentration $N_{A}$ increased, the built-in electric field increased; which resulted in separation of charge carriers and, hence, led to an increased $V_{\mathrm{oc}}$ and $J_{\mathrm{sc}}$ and improved solar cell performance [58].

However, as doping concentration continued to increase and exceeded $N_{A}=6.3\times 10^{16}{\;\mathrm{cm}}^{-3}$, scattering increased and, hence, carriers were no longer efficiently collected and recombination rates increased significantly, and all photovoltaic performance parameters showed a downward trend. Thus, further increase of the doping concentration was not favorable.

In conclusion, it was found in this part that $N_{t}=10^{14}$ and$\;10^{15}{\;\mathrm{cm}}^{-3}$ along with $N_{A}=6.3\times 10^{16}{\;\mathrm{cm}}^{-3}$ led to almost the same values of the PSC parameters. It is well known that a lower value of $N_{t}$ induces a higher fabrication cost; therefore, in the upcoming parts, optimal values of $N_{t}=10^{15}{\;\mathrm{cm}}^{-3}$ and $N_{A}=6.3\times 10^{16}{\;\mathrm{cm}}^{-3}$ were considered.

### 3.3. Effect of ETL/Perovskite and Perovskite/HTL Defect Interface on the Solar Cell Performance

According to [2,72], the interface defect density plays a major role in determining the performance of the PSC. Hence, this section is dedicated to the study of the impact of interface defect density in two scenarios: on one hand, at the ETL/PSC interface and, on the other hand, at the PSC/HTL interface.

**Figure 8:**
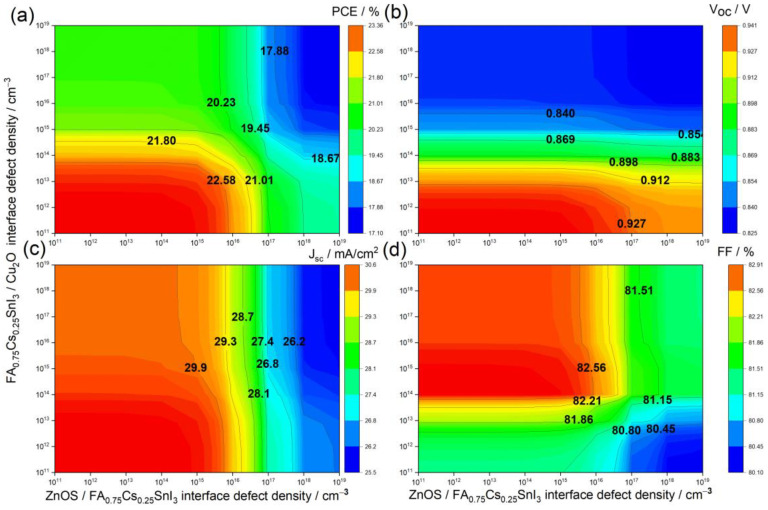
Solar Cell characteristics as function of interface defect densities. (**a**) PCE, (**b**) $V_{\mathrm{oc}}$, (**c**) $J_{\mathrm{sc}}$, and (**d**) FF.

Figure 8 illustrates the variation of the PSC parameters (PCE, $V_{\mathrm{oc}}$, $J_{\mathrm{sc}}$, and FF) as function of ETL/PSC interface defect density (*x*-axis) and PSC/HTL interface defect density (*y*-axis) both between $10^{11}$ and $10^{19}{\;\mathrm{cm}}^{-3}$. It can be generally noticed from Figure 8a that the PCE decreased from 24% to 17.1% with both interface defect densities. $V_{\mathrm{oc}}$ presented a similar behavior, but with much less dependency on the PSC/ETL defect density. Conversely, $J_{\mathrm{sc}}$ decreased with PSC/ETL defect density, with weaker dependency on HTL/PSC defect density. It is worth mentioning here that defect density at ETL strongly affected the $J_{\mathrm{sc}},$ since light enters from the ETL layer and most of the carrier generation occurred close to this interface.

Regarding FF, at PSC/HTL defect densities below $10^{16}{\;\mathrm{cm}}^{-3}$ FF abruptly increased with PSC/HTL defect density and, then, slightly decreased (less than 1.5%). This change in behavior occurred at a PSC/HTL defect density of approximately $5\times 10^{14}{\;\mathrm{cm}}^{-3}$. For ETL/PSC defect densities higher than$\;10^{16}{\;\mathrm{cm}}^{-3}$, FF would slightly increase with ETL/PSC defect density, until it reached a constant value of 81.2% at an ETL/PSC defect density of almost$\;10^{14}{\;\mathrm{cm}}^{-3}$.

Indeed, the lower the interface defects densities, the better the PSC performance was. However, taking into consideration the high cost of fabrication of devices with such low interface defect densities, it is, hence, necessary to adopt the lowest pair of defect densities leading to the best PSC performance and fabrication cost. Therefore, based on the above analysis, the optimal values of PSC/HTL and ETL/PSC defect densities were found to be $10^{13}$ and$\;10^{16}{\;\mathrm{cm}}^{-3}$, respectively; resulting in: PCE =22.58%,${\;V}_{\mathrm{oc}}=0.927\;V$, $J_{\mathrm{sc}}=29.9\;\mathrm{mA}/{\mathrm{cm}}^{2}$, and FF=81.86%.

### 3.4. Effect of Absorber Layer (FA_75_Cs_25_SnI_3_) Thickness

The thickness of the light-absorbing layer was found to be of high importance to the solar cell performance. The choice of thickness is delicate. A large value maximizes current density, but minimizes the reverse saturation current, all while increasing the fabrication cost. This section is dedicated to the study of the impact of absorber layer thickness on device photovoltaic outputs. In this study, the studied absorber layer was FA_75_Cs_25_SnI_3_, with different thicknesses varying up to 2.1 μm, while maintaining constant all the other parameters given in Table 1.

**Figure 9:**
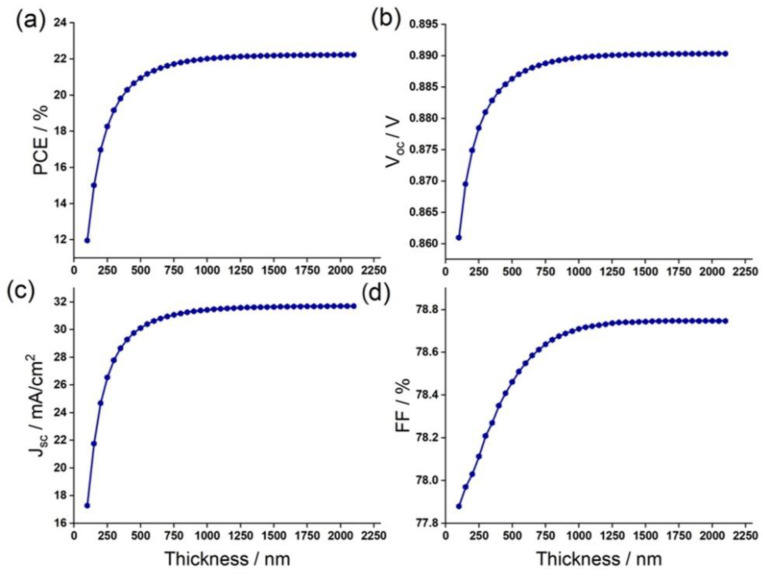
Change in (**a**) PCE, (**b**)${\;V}_{\mathrm{oc}}$, (**c**) $J_{\mathrm{sc}}$ and (**d**) FF against the FA_75_Cs_25_SnI_3_ absorber layer thickness.

Figure 9 depicts the variation of PSC properties $\mathrm{PCE},{\;V}_{\mathrm{oc}}$, $J_{\mathrm{sc}}$, and FF with the absorber thickness. It can be observed that $\mathrm{PCE},{\;V}_{\mathrm{oc}}$, $J_{\mathrm{sc}}$ showed the same behavior: they drastically increased when the absorber was thin, then they became saturated when the thickness reached 1 μm. Above this value, the effect of absorber layer thickness became minimal. For this thickness, PSC showed a PCE of 22%, $V_{\mathrm{oc}}=0.89\;V$ and $J_{\mathrm{sc}}=31.8\;\mathrm{mA}/{\mathrm{cm}}^{2}$. The great enhancement of $J_{\mathrm{sc}}$ with increase of the absorber thickness was related to the generation of more electron-hole pairs in the perovskite, leading to an efficiency enhancement. Regarding the FF, it could be noticed that the values decreased from 80.4% to 78.25% when the absorber thickness was of 0.1 and 0.3 μm, respectively. Then, FF increased and reached a value of 78.75% at a thickness of 1 μm, before saturating.

**Figure 10:**
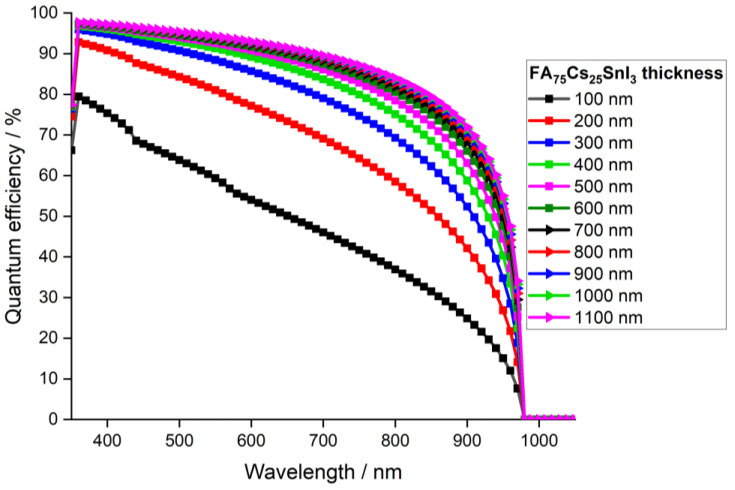
Quantum efficiency of the simulated device with absorber layer thickness.

Figure 10 represents the quantum efficiency as a function of the light wavelength for various absorber thicknesses, ranging from 0.1 to 1.1 μm.

Quantum efficiency (QE) indicates the capability of a solar cell to collect carriers from incident photons of a given energy/wavelength. From Figure 10, it can be noticed that when the absorber thickness increased, quantum efficiency increased, indicating that the photon absorption at longer wavelength was enhanced. This fact was due to the large number of photogenerated electron–hole pairs inside the absorber layer. In addition, at wavelengths larger than 980 nm, quantum efficiency fell to zero, as light was not absorbed below the bandgaps at longer wavelengths (lower energy). The highest QE was reached when the absorber thickness was 1 μm. Afterwards, at higher thicknesses, the curves overlapped, indicating a saturation in QE values. Therefore, Figure 10 confirms through quantum efficiency, that an absorber of thickness 1 μm was sufficient to obtain an optimal device.

## 4. Conclusions

In this study, a robust and stable ${\mathrm{FA}}_{1-x}{\mathrm{Cs}}_{x}{\mathrm{SnI}}_{3}$-based perovskite solar cell was studied and compared to a pure FASnI_3_-based PSC. A normal n-i-p planar structure of FTO/ETL/${\mathrm{FA}}_{1-x}{\mathrm{Cs}}_{x}{\mathrm{SnI}}_{3}$/Cu_2_O/Au was numerically simulated and investigated using SCAPS-1D simulation software. The effect of TiO_2_ and ZnOS ETL on the solar cell performance was thoroughly investigated. The study proved that solar cells with ZnOS have outstanding performance, due to the high electron mobility in the ZnOS layer and excellent band alignment of ZnOS against all tested perovskites with different Cs contents.

The CBO between the ETL and the perovskite was mainly affected by the Cs content in the perovskite. The work herein clearly explained the significant effect of Cs doping and CBO on the electrical performance of the cells. The solar cells with pure FASnI_3_ as absorber, had, by far, the best performance among all the PSCs with TiO_2_ ETL. However, solar cells with ${\mathrm{FA}}_{75}{\mathrm{Cs}}_{25}{\mathrm{SnI}}_{3}$ as absorber and ZnOS as ETL outperformed all the simulated devices.

Furthermore, the performance of the latter device was optimized by tuning four major factors: doping concentration $N_{A}$ and defect density $N_{t}$ of the absorber layer, ${\mathrm{FA}}_{75}{\mathrm{Cs}}_{25}{\mathrm{SnI}}_{3}$ absorber layer thickness, and the defect concentration at ETL/perovskite and perovskite/HTL interfaces. The results revealed that the ultimate device FTO/ ZnOS /${\mathrm{FA}}_{75}{\mathrm{Cs}}_{25}{\mathrm{SnI}}_{3}$/Cu_2_O/Au was obtained with the following factors: absorber defect density $N_{t}=10^{15}{\;\mathrm{cm}}^{-3}$, absorber doping concentration $N_{A}=6.3\times 10^{16}{\;\mathrm{cm}}^{-3}$, and light absorber thickness of 1 μm. The optimal values of PSC/HTL and ETL/PSC defect densities were $10^{16}{\;\mathrm{cm}}^{-3}$ and$\;10^{13}{\;\mathrm{cm}}^{-3}$, respectively. Minimizing the absorber defect density and the defect densities at the interface greatly improved the PCE to reach an unprecedented result of almost 22%. Thus, future studies should be devoted to refining the device deposition methods. The novel results obtained clearly show a possible way to fabricate cost-effective, highly efficient, and stable ${\mathrm{FA}}_{75}{\mathrm{Cs}}_{25}{\mathrm{SnI}}_{3}$-based perovskite solar cells.

## Figures and Tables

**Table 1:** Electrical and optical properties used in simulation of ${\mathrm{FA}}_{1-x}{\mathrm{Cs}}_{x}{\mathrm{SnI}}_{3}$ -based perovskite solar cell.

Parameters	FTO (TCO) [55]	FASnI_3_ [19,56,57]	FA_90_Cs_10_SnI_3_ [19,56,57]	FA_85_Cs_15_SnI_3_ [19,56,57]	FA_75_Cs_25_SnI_3_ [19,56,57]
Thickness/µm	0.4	0.45	0.45	0.45	0.45
Bandgap $E_{g}$/eV	3.5	1.45	1.35	1.33	1.27
Electron Affinity χ/eV	4.3	4	3.92	3.65	3.8
Dielectric permittivity	9	8.2	8.2	8.2	8.2
CB effective density of states/${\mathrm{cm}}^{-3}$	$2.2\times 10^{18}$	$1\times 10^{18}$	$1\times 10^{18}$	$1\times 10^{18}$	$1\times 10^{18}$
VB effective density of states/${\mathrm{cm}}^{-3}$	$1.8\times 10^{19}$	$1\times 10^{18}$	$1\times 10^{18}$	$1\times 10^{18}$	$1\times 10^{18}$
Electron mobility/ ${\mathrm{cm}}^{2}/V\cdot s$	20	22	22	22	22
Hole mobility/ ${\mathrm{cm}}^{2}/V\cdot s$	10	22	22	22	22
Donor Concentration $N_{D}$/ ${\mathrm{cm}}^{-3}$	$1\times 10^{18}$	0	0	0	0
Acceptor concentration $N_{A}$/${\mathrm{cm}}^{-3}$	0	$7\times 10^{16}$	$7\times 10^{16}$	$7\times 10^{16}$	$7\times 10^{16}$

**Table 2:** Electrical and optical properties of different ETL and HTL materials.

Parameters	Cu_2_O (HTL) [62,63]	TiO_2_ (ETL) [58,61]	ZnOS (ETL) [64]
Thickness/µm	0.350	0.05	0.05
$\mathrm{Bandgap}\;E_{g}$/eV	2.170	3.260	2.83
Electron Affinity χ/eV	3.2	4	3.6
Dielectric permittivity	7.11	32	9
CB effective density $\mathrm{of}\;\mathrm{states}/{\mathrm{cm}}^{-3}$	$2.02\times 10^{17}$	$1\times 10^{19}$	$2.2\times 10^{18}$
VB effective density of $\mathrm{states}/{\mathrm{cm}}^{-3}$	$1.1\times 10^{19}$	$1\times 10^{19}$	$1.8\times 10^{19}$
Electron mobility/ ${\mathrm{cm}}^{2}/V\cdot s$	20	20	100
Hole mobility/ ${\mathrm{cm}}^{2}/V\cdot s$	80	10	25
Donor Concentration $N_{D}/\;{\mathrm{cm}}^{-3}$	$1\times 10^{7}$	$1\times 10^{17}$	$1\times 10^{17}$
$\mathrm{Acceptor}\;\mathrm{concentration}\;N_{A}/{\mathrm{cm}}^{-3}$	$1\times 10^{18}$	0	0

**Table 3:** Defect density values inside the layers and at interface of the device.

Parameters	ETL	HTL	$FA_{1-x}Cs_{x}SnI_{3}$	$\mathrm{HTL}/FA_{1-x}Cs_{x}SnI_{3}$	$FA_{1-x}Cs_{x}SnI_{3}/\mathrm{ETL}$
Defect Type	Neutral	Neutral	Neutral	Neutral	Neutral
Capture cross section $\mathrm{for}\;\mathrm{electrons}\;\sigma_{n}/{\mathrm{cm}}^{-2}$	$1\times 10^{-15}$	$1\times 10^{-15}$	$1\times 10^{-15}$	$1\times 10^{-18}$	$1\times 10^{-15}$
Capture cross section $\mathrm{for}\;\mathrm{hole}\;\sigma_{p}/{\mathrm{cm}}^{-2}$	$1\times 10^{-15}$	$1\times 10^{-15}$	$1\times 10^{-15}$	$1\times 10^{-16}$	$1\times 10^{-15}$
Energetic distribution	Single	Single	Gaussian	Single	Single
$\mathrm{Energy}\;\mathrm{level}\;\mathrm{with}\;\mathrm{respect}\;\mathrm{to}\;E_{v}$$\;(\mathrm{above}\;E_{v}$)/eV	0.6	0.650	0.6	0.6	0.6
Characteristic energy/eV	0.1	0.1	0.1	0.1	0.1
$\mathrm{Total}\;\mathrm{density}\;N_{t}/{\mathrm{cm}}^{-3}$	$1\times 10^{15}$	$1\times 10^{15}$	$1\times 10^{15}$	$1\times 10^{12}$	$1\times 10^{11}$

**Table 4:** CBO for ETL ZnOS and TiO_2_ per Cs content.

Cs Content/%	CBO ETL ZnOS/eV	CBO ETL TiO_2/_eV
0	0.4	0
10	0.32	−0.08
15	0.05	−0.35
25	0.2	−0.2

## Data Availability

Not applicable.
